# Inhibition of Diacylglycerol Lipase Impairs Fear Extinction in Mice

**DOI:** 10.3389/fnins.2018.00479

**Published:** 2018-07-31

**Authors:** Victoria S. Cavener, Andrew Gaulden, Dante Pennipede, Puja Jagasia, Jashim Uddin, Lawrence J. Marnett, Sachin Patel

**Affiliations:** ^1^Department of Psychiatry and Behavioral Sciences, Vanderbilt University Medical Center, Nashville, TN, United States; ^2^Vanderbilt Brain Institute, Vanderbilt University, Nashville, TN, United States; ^3^Departments of Biochemistry, Chemistry, and Pharmacology, A.B. Hancock Jr. Memorial Laboratory for Cancer Research, Vanderbilt Institute of Chemical Biology, Vanderbilt University School of Medicine, Nashville, TN, United States

**Keywords:** FAAH, endocannabinoid, fear, stress, extinction, cannabinoid, 2-arachidonoylglycerol

## Abstract

Elucidating the underlying molecular mechanisms regulating fear and extinction learning may offer insights that can lead to novel treatments for debilitating anxiety and trauma-related disorders including posttraumatic stress disorder. The endocannabinoid (eCB) system is a retrograde inhibitory signaling pathway involved in regulating central responses to stress. The eCB 2-arachidonoylglycerol (2-AG) has recently been proposed to serve as a homeostatic signal mitigating adverse effects of stress exposure, however, less well understood is 2-AG’s role in fear learning and fear extinction. In this study, we have sought to explore 2-AG’s role in fear conditioning and fear extinction by disrupting 2-AG synthesis utilizing the DAGL inhibitor (DO34) and DAGLα knock-out mice (DAGLα^−/−^). We found that DAGLα^−/−^ mice, and male and female C57B6/J mice treated with DO34, exhibited impairment in extinction learning in an auditory cue fear-conditioning paradigm. DO34 did not increase unconditioned freezing. Interestingly, inhibition of fatty-acid amide hydrolase was not able to restore normal fear extinction in DO34-treated mice suggesting increased Anandamide cannot compensate for deficient 2-AG signaling in the regulation of fear extinction. Moreover, augmentation of CB1R signaling with tetrahydrocannabinol also failed to restore normal fear extinction in DO34-treated mice. Overall, these data support the hypothesis that DAGLα plays an important role in fear extinction learning. Although genetic and pharmacological disruption of DAGL activity causes widespread lipidomic remodeling, these data combined with previous studies putatively suggest that deficient 2-AG signaling could be a susceptibility endophenotype for the development of trauma-related psychiatric disorders.

## Introduction

Over the past 25 years, studies have shown that the endocannabinoid (eCB) system is a key regulator of an organism’s response to stress and plays an important role in facilitating recovery after exposure to stress (Lutz et al., 2015; Patel et al., 2017; Hill et al., 2018). Dysregulation of fear learning, fear extinction learning, and the abnormal retention of a heightened fear response have been implicated in many anxiety-related mental illnesses including posttraumatic stress disorder (PTSD) (Parsons and Ressler, 2013; Maren and Holmes, 2016; Deslauriers et al., 2017). Understanding the molecular mechanisms involved in fear learning, fear extinction, and the development of generalized anxiety with persistent hyper responsiveness to stressful situations, could lead to important insights into the pathophysiological mechanisms underlying fear adaptations and potentially novel treatment approaches for stress-related mental disorders. In this study, we investigate the role of eCBs in fear learning and fear extinction via pharmacological and genetic modulation of 2-arachidonoylglycerol (2-AG) synthesis in male and female mice.

The eCB system is a retrograde inhibitory signaling pathway composed of the presynaptic cannabinoid CB1 receptor (CB1R) and its endogenous ligands arachidonoylethanolamine (AEA) and 2-AG (Herkenham et al., 1990; Matsuda et al., 1990; Devane et al., 1992; Piomelli, 2003; Ohno-Shosaku and Kano, 2014). 2-AG is the most abundant eCB in the brain and is acutely increased by stress exposure (Kondo et al., 1998; Patel et al., 2005, 2009; Dubreucq et al., 2012; Bedse et al., 2017). It is hypothesized that this stress-induced increase in 2-AG signaling serves to counteract some of the adverse behavioral consequences of stress exposure (Hohmann et al., 2005; Evanson et al., 2010; Hill et al., 2011; Wang et al., 2012; Bluett et al., 2014, 2017; Bedse et al., 2017). Conversely, AEA is decreased in response to stress, and plays a role in activating the stress response within the HPA-axis (Dubreucq et al., 2012; McLaughlin et al., 2012; Wang et al., 2012; Gray et al., 2015, 2016). Studies have shown that augmenting 2-AG reduces stress-induced anxiety-like and depressive-like behaviors and can promote resilience to the adverse effects of acute and repeated stress (Kinsey et al., 2011; Sciolino et al., 2011; Sumislawski et al., 2011; Roberts et al., 2014; Zhang et al., 2015; Bedse et al., 2017; Bluett et al., 2017; Heinz et al., 2017). 2-AG augmentation can also increase active fear responses to threats (Heinz et al., 2017). Surprisingly, 2-AG augmentation promotes the expression of conditioned freezing and impairs conditioned fear extinction learning (Llorente-Berzal et al., 2015; Hartley et al., 2016). This surprising contradiction highlights the complexity of eCB signaling in the brain and motivated us to further investigate the role of 2-AG signaling in the regulation of fear learning and extinction.

The DAGLα is a key enzyme responsible for 2-AG synthesis in the postsynaptic neuron in response to increased synaptic activity (Bisogno et al., 2003; Tanimura et al., 2010; Shonesy et al., 2014). Repeated homotypic stress results in increased 2-AG production via DAG hydrolysis by DAGLα (Patel et al., 2009). DAGLα^−/−^ mouse models show reduced levels of 2-AG [and in some cases AEA (Tanimura et al., 2010; Jenniches et al., 2016)], increases in anxiety associated behaviors and increased susceptibility to adverse behavioral consequences of stress exposures (Shonesy et al., 2014; Jenniches et al., 2016; Bluett et al., 2017). These studies are consistent with work demonstrating reducing 2-AG levels via MAGL overexpression also increases anxiety-like behaviors (Guggenhuber et al., 2015). In addition, acute treatment with the DAGL inhibitor DO34 increases innate anxiety levels and promotes susceptibility to the adverse behavioral consequences of stress in mice (Bedse et al., 2017; Bluett et al., 2017). Interestingly, DAGLα^−/−^ also exhibit impairment in extinction of conditioned fear responses (Jenniches et al., 2016). Here, we aim to replicate and extend these findings to gain insight into how DAGL regulates fear extinction learning using a combination of pharmacological and genetic approaches in male and female mice. Overall, our convergent pharmacological and genetic data demonstrate an important role for DAGL in the regulation of fear extinction and further suggest deficient 2-AG-mediated eCB signaling may be an important susceptibility endophenotype subserving risk for the development of trauma-related psychiatric disorders.

## Materials and Methods

### Animals

The 9- to 12-week-old male and female C57BL/6J were used as subjects (Jackson Laboratory, Bar Harbor, ME, United States). Male and female DAGLα^−/−^ mice on a C57BL/6J background were bred in house as described previously and used in one experiment (Shonesy et al., 2014; Bluett et al., 2017). All mice were habituated for one week at Vanderbilt Murine Neurobehavior Core prior to behavior testing. Three to five mice were housed per cage in a temperature and humidity controlled housing facility under a 12-h light/dark cycle, with access to food and water *ad libitum*. Behavior experiments were performed during dark cycle under red light. All studies were approved by the Vanderbilt University Animal Care and Use Committees, and were conducted in accordance with the National Institute of Health Guide for the Care and Use of Laboratory Animals.

### Drug Treatment

Mice were given an intraperitoneal (IP) injection of DO34 (50 mg/kg) synthesized as previously described (Ogasawara et al., 2016) dissolved in an 18:1:1 solution of saline, ethanol, and kolliphor EL (Sigma–Aldrich, St. Louis, MO, United States), or vehicle alone (18:1:1 solution of saline, ethanol and kolliphor EL) 2 h prior to behavioral testing at a volume of 10 ml/kg. In one experiment, mice were treated with a combination of DO34 (50 mg/kg) and PF-3845 (FAAH Inhibitor) at 1 mg/kg (A gift from Pfizer Central Research), via IP injection 2 h prior to behavioral testing, or just DO34 (50 mg/kg) alone. In two experiments, mice were treated with a combination of IP injection of DO34 (50 mg/kg) 2 h prior to extinction training and Tetrahydrocannabinol (THC; CB1R partial agonist) at 0.3 mg/kg (in one experiment) or 0.6 mg/kg (Cayman Chemical Company-18:1:1 solution of saline, ethanol and kolliphor EL), via IP injection 30 min prior to behavioral testing, or just DO34 (50 mg/kg) alone. Doses utilized in this study were similar to those previously described in (Bedse et al., 2017).

### Fear-Condition and Extinction Paradigm

Freezing behavior was measured using video analysis software (Video Freeze-Med Associates) during all of the fear conditioning and extinction trials exactly as described previously (Hartley et al., 2016). The mice were placed in an auxiliary room directly adjacent to the testing room and allowed to acclimate for 30 min prior to each trial under red light, with consistent temperature and humidity conditions between the auxiliary and trial room. At the end of each protocol, the mice were placed in their respective home cages in the auxiliary room and later returned to the housing facility.

On testing day 1, the mice were placed in a square Plexiglas chamber (dimensions: 30.5 × 24.1 × 21.0 cm) housed in a sound proofing box developed by Med. Associates. Context A consists of a bare metal grid floor, no insert along the walls, and no added scent. Baseline measurements were taken for 90 s. After the baseline, six 30-s tones – conditioned stimulus (CS), were played through a chamber wall mounted speaker, each tone was followed by followed by a 2-s 70 mA shock (unconditioned stimulus-US). There was a 30-s delay between each tone. All mice who failed to freeze at least 50% by final CS on conditioning day were removed from analysis except in **Figure 2** where effects of DO34 on conditioning *per se* were examined.

On testing days 2 and 3, mice were placed in the conditioning chamber in Context B for extinction training. Context B has a smooth white plastic floor insert to cover the metal grid, as well as a curved plastic insert along the side and back walls made of the same white plastic material as the floor. The wall insert has perforations that align with the wall mounted speaker in order to ensure the sound quality of the (CS). Additionally, a paper towel soaked in vanilla extract was placed under the floor insert and grid as a novel olfactory queue, specific to Context B. A baseline measure of freezing behavior was recorded for 30 s followed by a series of 20 30-s tones with a 30-s delay between each tone. No shock was administered during the extinction training.

On day 4, the mice were put back in Context B for CS recall. No drug was administered prior to CS recall. Baseline measure was taken for 2.5 min followed by five 30-s tones with a 30-s delay between each tone. A final measure of extinction recall was taken after the final tone for 2.5 min.

### Statistical Analysis

The freezing data for each group was analyzed using a repeated measures two-way ANOVA factoring trial block (time) and drug treatment/or genotype. Total freezing time was entered as reported by the video analysis software. All statistical analyses were conducted using Prism GraphPad 7. *P* < 0.05 was considered significant throughout. *F*- and *P*-values for significant effects of drug treatment or genotype can be found within figure panels. Effect size was calculated using formula for η^2^ to reflect the proportion of total variability in the freezing behavior that is accounted for by variation in genotypes or drug treatment during each trial (Tabachnick and Fidell, 2007).

## Results

### DAGLα^−/−^ Mice Have Impaired Fear Extinction

**FIGURE 1:**
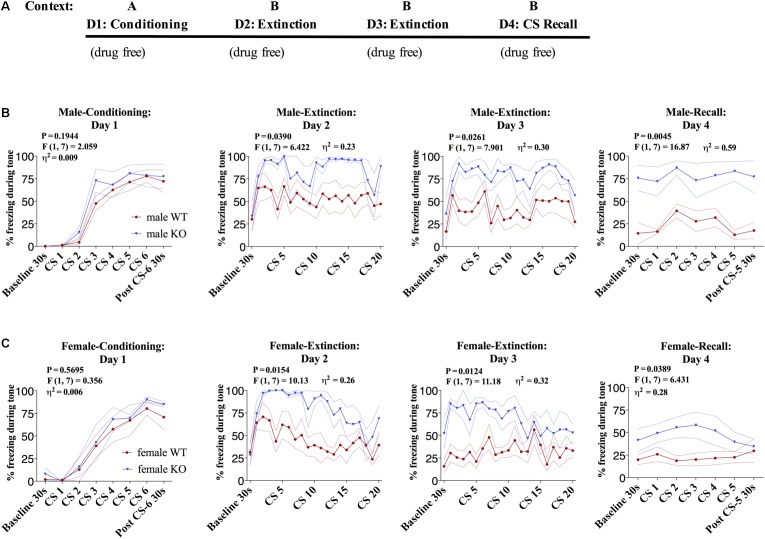
Male and female DAGLα^−/−^ mice have impaired fear extinction. **(A)** Schematic diagram of the experimental paradigm. **(B)** (Far left panel) % freezing by male DAGLα^−/−^ and WT mice during acquisition of cue-conditioned fear. (Middle panels) % freezing during auditory cue during extinction training days 2 and 3. (Far right panel) % freezing during extinction recall (*n* = 5 WT, *n* = 4 KO mice). **(C)** (Far left panel) % freezing by female DAGLα^−/−^ and WT mice during acquisition of cue-conditioned fear. (Middle panels) % freezing during auditory cue during extinction training days 2 and 3. (Far right panel) % freezing during extinction recall (*n* = 5 WT, *n* = 4 KO mice). *F*- and *P*-values and η^2^ for genotype effect shown in each panel. All values are presented as mean ± SEM.

Given that it has been previously shown that global DAGLα^−/−^ mice exhibit impaired fear extinction (Jenniches et al., 2016), we first aimed to replicate these findings in our line of global DAGLα^−/−^ mice in a fear conditioning and extinction protocol we and others have previously utilized extensively (Hartley et al., 2016) (**Figure 1A**). There was no significant difference in freezing to tone during fear-conditioning on day 1 between DAGLα^−/−^ and WT littermate controls (**Figures 1B,C**). However, freezing behavior in response to tone presentation during extinction was increased in both male and female DAGLα^−/−^ mice compared to WT littermate controls [**Figures 1B,C**, female day 2 and 3: *P* = 0.0154/0.0124, *F*(1,7) = 6.422/11.18, η^2^= 0.23/0.32, males day 2 and 3: (*P* = 0.0390/0.0261, *F*(1,7) = 10.13/7.901, η^2^= 0.26/0.30)]. Freezing levels were also significantly higher during extinction recall in DAGLα^−/−^ male and female mice compared to WT mice during both baseline and tone presentation [females day 4:*P* = 0.0389, *F*(1,7) = 6.431, η^2^= 0.28, males day 4: *P* = 0.0045, *F*(1,7) = 16.87, η^2^= 0.59].

### Pharmacological DAGL Inhibition Does Not Affect Acquisition of Conditioned-Fear

**FIGURE 2:**
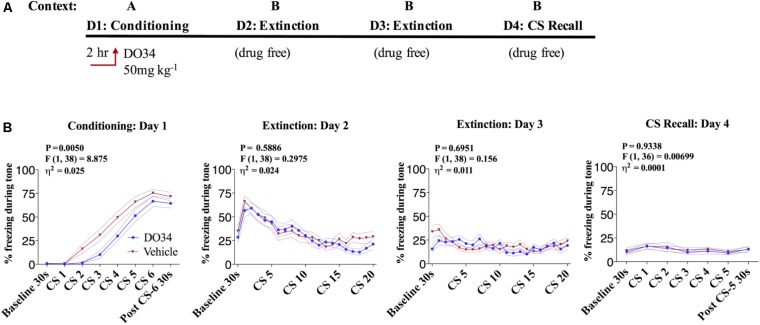
Pharmacological DAGL inhibition does not affect acquisition of conditioned-fear. **(A)** Schematic diagram of the experimental paradigm. **(B)** (Far left panel) % freezing by C57BL/6J male mice during acquisition of cue-conditioned fear when DO34 (50 mg kg^−1^) was injected IP 2 h prior to trial. (Middle panels) % freezing during auditory cue during extinction training days 2 and 3. (Far right panel) % freezing during extinction recall (*n* = 20 male mice per condition). *F*- and *P*-values and η^2^ for main effect of drug treatment show in each panel. All values are presented as mean ± SEM.

In order to confirm the effects observed in DAGLα^−/−^ mice were mediated via impaired enzymatic activity during adulthood and to gain insight into the temporal regulation of fear learning and extinction by DAGL, we utilized a pharmacological inhibitor of DAGL, DO34 (Ogasawara et al., 2016). We first tested whether acquisition of conditioned fear was regulated by DAGL activity via administration of DO34 or vehicle 2 h prior to fear conditioning on day one in Context A (**Figure 2A**). We observed a very small but significant decrease in freezing behavior in DO34-treated male mice compared to vehicle-treated controls during conditioning [**Figure 2B**, day 1: *P* = 0.050, *F*(1,38) = 8.875, η^2^= 0.025] suggesting a slight delay in the acquisition of conditioned freezing behavior. In contrast, there was no significant difference in subsequent freezing behavior during tone presentation during extinction training on days 2–3 nor during extinction recall on day 4 in the drug-free states.

### Pharmacological DAGL Inhibition Impairs Fear Extinction

**FIGURE 3:**
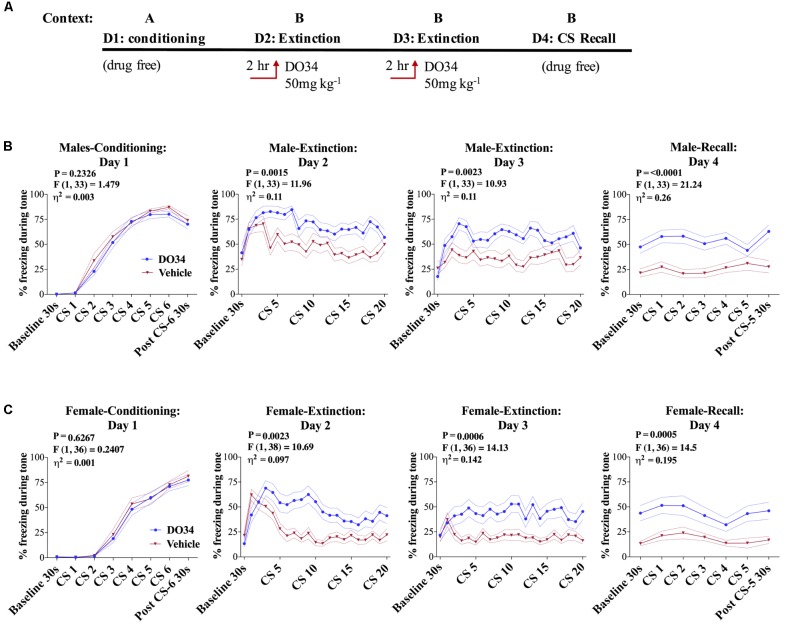
Pharmacological DAGL inhibition impairs fear extinction. **(A)** Schematic diagram of the experimental paradigm. **(B)** (Far left panel) % freezing by C57BL/6J male mice during acquisition of cue-conditioned fear. (Middle panels) % freezing during auditory cue by mice during extinction training days 2 and 3 when D034 (50 mg kg^−1^) was injected IP 2 h prior to trial. (Far right panel) % freezing during extinction recall (*n* = 17 DO34-treated male mice, *n* = 18 vehicle-treated male mice). **(C)** (Far left panel) % freezing by C57BL/6J female mice during acquisition of cue-conditioned fear when DO34 (50 mg kg^−1^) was injected IP 2 h prior to trial. (Middle panels) % freezing during auditory cue during extinction training days 2 and 3. (Far right panel) % freezing during extinction recall (*n* = 18 DO34-treated female mice *n* = 20 vehicle-treated female mice). *F*- and *P*-values and η^2^ for main effects of drug treatment shown in each panel. All values are presented as mean ± SEM.

Data derived from global DAGLα^−/−^ mice suggest a potential role for 2-AG signaling in the regulation of fear extinction; however, limitations of global knock-out models make conclusive interpretations in this regard difficult (Jenniches et al., 2016). To circumvent these limitations, we next determined whether acute depletion of 2-AG using the pharmacological DAGL inhibitor DO34 would also inhibit fear extinction learning in male and female mice (**Figure 3A**). We utilized a dose of 50 mg/kg which we have shown causes a near-complete elimination of measurable 2-AG throughout the brain (Bedse et al., 2017; Bluett et al., 2017). Prior to conditioning, mice were assigned to vehicle or DO34 treatment groups. There were no differences in freezing response to tone-sock pairings between groups (**Figure 3B**), confirming similar conditioning efficiency in both treatment groups. On day two, DO34 or vehicle was injected 2 h prior to extinction training. DO34-treated male and female mice showed a significant increase in percent freezing time during tone presentation on both extinction day 2 and extinction day 3 [**Figures 3B,C**, day 2 and 3: female *P* = 0.0023/0.0006, *F*(1,38) = 10.69/14.13, η^2^= 0.097/0.142, male *P* = 0.0015/0.0023, *F*(1,33) = 11.96/10.93, η^2^= 0.11/0.11]. During the extinction recall test on day 4, performed under drug-free conditions, previously DO34-treated mice showed a sensitized freezing response during baseline and subsequent tone presentation relative to vehicle-treated mice [**Figures 3B,C**, day 4: female *P* = 0.0005, *F*(1,36) = 14.5, η^2^= 0.195, male *P* = 0.0015, *F*(1,33) = 11.96, η^2^= 0.11]. Similar effects were observed in female mice (**Figure 3C**). The effect of DO34 treatment during extinction training was similar to that observed in mice that did not undergo extinction training compared to those that did [**Supplementary Figure S1**, day 4: *P* = 0.0075, *F*(1,28) = 8.136, η^2^= 0.166], indicating DAGL inhibition during extinction training reduces the effectiveness of fear extinction training, mirroring effects obtained in mice which had not undergone extinction training at all.

### Pharmacological DAGL Inhibition Does Not Affect Unconditioned Freezing

**FIGURE 4:**
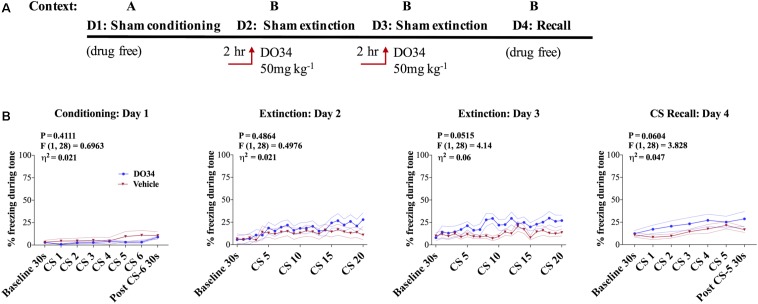
Pharmacological DAGL inhibition does not affect unconditioned freezing. **(A)** Schematic diagram of the experimental paradigm. **(B)** (Far left panel) % freezing by C57BL/6J male mice during sham-conditioning assay-no shock was administered after tone. (Middle panels) % freezing during auditory cue during extinction training days 2 and 3. (Far right panel) % freezing during extinction recall (*n* = 15 male mice per condition). *F*- and *P*-values and η^2^ for main effect of drug treatment shown in each panel. All values are presented as mean ± SEM.

Given that DAGLα inhibition can increase unconditioned anxiety (Shonesy et al., 2014; Jenniches et al., 2016), we wanted to rule out the possibility that DO34 increased freezing behavior independent of a fear-conditioning. To explicitly test this, mice were tested in a sham conditioning paradigm where, on day one in Context A, mice were exposed to tone without successive shocks (i.e., CS only). On days 2–3, mice were injected with DO34 2 h prior to sham fear extinction training (**Figure 4A**). Freezing behavior was not different between DO34-treated and vehicle-treated mice on days 2–3 of sham extinction, or on day 4 of sham extinction recall (**Figure 4B**). These data indicate that DO34 does not increase freezing behavior independent of conditioning.

### AEA Augmentation Does Not Reverse Impaired Extinction After DAGL Inhibition

**FIGURE 5:**
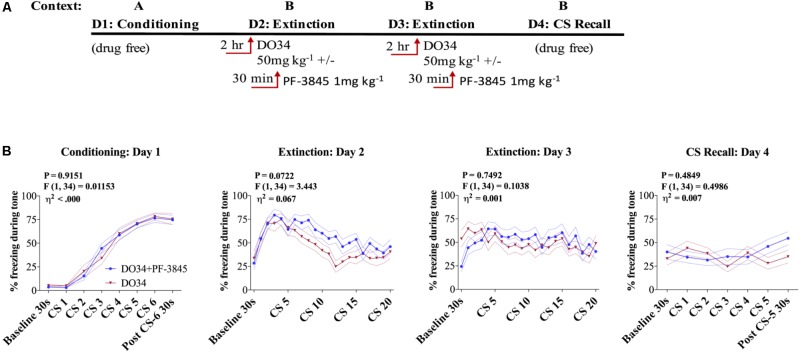
AEA augmentation does not reverse impaired extinction after DAGL inhibition. **(A)** Schematic diagram of the experimental paradigm. **(B)** (Far left panel) % freezing by C57BL/6J male mice during acquisition of cue-conditioned fear. (Middle panels) % freezing during auditory cue by mice during extinction training days 2 and 3 when DO34 (50 mg kg^−1^) was injected alone or in addition to PF-3845 (FAHH inhibitor 1 mg kg^−1^) IP 2 h prior to trial. (Far right panel) % freezing during extinction recall (*n* = 18 male mice per condition). *F*- and *P*-values and η^2^ for main effect of drug shown in each panel. All values are presented as mean ± SEM.

In order to determine whether augmentation of AEA signaling could compensate for deficient 2-AG synthesis and promote successful fear extinction, we blocked AEA degradation using the FAAH inhibitor PF-3845 concomitantly with DO34 treatment during extinction training (**Figure 5A**). We found no significant difference between the groups’ freezing behaviors when mice were given DO34 + FAAH Inhibitor or DO34 alone, prior to fear extinction training on days 2 or 3 (**Figure 5B**).

### CB1R Partial Agonist THC Does Not Reverse Impaired Extinction After DAGL Inhibition

In order to determine whether activation of CB1R receptors could compensate for deficient 2-AG synthesis and promote successful fear extinction, we injected THC (0.3–0.6 mg/kg) 30 min before extinction training on days 2 and 3. We found no significant difference between the groups’ freezing behaviors when mice were given DO34 + THC or DO34 alone, prior to fear extinction training on days 2 or 3 (**Supplementary Figure S2**).

## Discussion

The eCB signaling is a well-established regulator of fear extinction, however, the role of 2-AG in the regulation of these processes has only recently been studied due to previously limited pharmacological and genetic tools to interrogate 2-AG signaling *in vivo* (Llorente-Berzal et al., 2015; Hartley et al., 2016; Jenniches et al., 2016). Generation of pharmacological tools for 2-AG augmentation and depletion (Ogasawara et al., 2016), and development of global and conditional DAGLα^−/−^ mice (Shonesy et al., 2014; Bluett et al., 2017), has significantly enhanced our ability to examine the role of 2-AG within multiple stress-related biological processes. In this context, we aimed to utilize convergent pharmacological and genetic modulation of DAGL to elucidate the role of 2-AG signaling in fear-learning and extinction. The main findings of the present study are that (1) global life-long DAGLα deletion results in impaired extinction of conditioned fear, (2) acute pharmacological DAGL inhibition impairs extinction of conditioned fear behavior, and (3) neither FAAH inhibition nor THC were able to correct these deficits in fear extinction caused by acute DAGL inhibition. These data provide further support for the notion that 2-AG deficiency states could predispose to the development of stress-related psychopathology and that pharmacological approaches aimed at counteracting this deficiency could represent novel approaches to the treatment of an array of anxiety and trauma-related disorders (Bedse et al., 2017; Bluett et al., 2017; Hill et al., 2018).

With regard to the effects of 2-AG modulation on acquisition of conditioned fear behaviors, our pharmacological data suggest 2-AG signaling may be important for aversive learning as DO34 administered prior to conditioning slowed the acquisition of conditioned freezing behavior. This finding is consistent with data showing accelerated acquisition of conditioned freezing in a trace fear conditioning paradigm after 2-AG augmentation with the monoacylglycerol lipase (MAGL) inhibitor JZL184 (Xu et al., 2014). These data suggest 2-AG signaling may be important for optimal cognitive function consistent with the proposed nootropic effects of CB1 receptor stimulation in aging (Bilkei-Gorzo et al., 2017). However, cognitive impairment of CB1 stimulation have also been demonstrated (Kruk-Slomka et al., 2017), suggesting a potentially complex and/or divergent roles of eCB signaling relative to exogenous cannabinoid agonist administration.

Our findings that both genetic and pharmacological inhibitions of DAGL impair fear extinction are consistent with a recent study showing impaired long-term fear extinction in DAGLα^−/−^ mice (Jenniches et al., 2016). Our studies extend these findings by demonstrating impaired fear extinction after acute pharmacological DAGL inhibition, suggesting findings in DAGLα^−/−^ mice are not a consequence of developmental abnormalities or compensatory adaptations resulting from life-long DAGLα deletion. We further show that the effects of DAGL inhibition are not confounded by increases in unconditioned freezing. Overall, these data are consistent with long-standing findings that blockade or genetic deletion of CB1 receptors robustly impairs fear extinction (see (Lutz, 2007; Ruehle et al., 2012; Rabinak and Phan, 2014; Hill et al., 2018) for review), supporting the notion that 2-AG depletion secondary to DAGL inhibition may be the cause of the behavioral effects observed here. However, DAGL inhibition causes widespread changes in lipidomic networks and thus lack of ability to conclusively ascribe 2-AG deficiency as causally related to the observed phenotypes remains a limitation of the work. Lastly, extinction impairing effects of DO34 were only observed when relatively high shock intensities were used for the US (0.7 mA), but not lower (0.4 mA) intensities (data not shown). This caused significant fear generalization as evidence by unconditioned freezing during the baseline period in all mice on days 2, 3, and 4. Whether the effects of DAGL inhibition are explicitly dependent on US intensity remains to be determined.

That acute pharmacological DAGL inhibition is associated with impaired extinction of conditioned fear is also globally consistent with the increased unconditioned anxiety and increased stress-susceptibility observed after DAGL inhibition and in DAGLα^−/−^ mice (Shonesy et al., 2014; Jenniches et al., 2016; Bedse et al., 2017; Bluett et al., 2017). These data are also consistent with the anxiolytic effects of 2-AG augmentation in a variety of unconditioned anxiety models and the ability or 2-AG augmentation to reduce, and promote resilience to, the adverse effects of stress exposure (Busquets-Garcia et al., 2011; Kinsey et al., 2011; Sciolino et al., 2011; Sumislawski et al., 2011; Zhang et al., 2015; Morena et al., 2016; Bluett et al., 2017). Taken together, these recent and compelling findings suggest 2-AG is an important signaling molecule involved in reducing unconditioned anxiety and the adverse effects of stress, and facilitating appropriate extinction of aversive memories. They also suggest 2-AG deficiency could represent a susceptibility endophenotype predisposing to anxiety and trauma-related psychiatric disorders (see Hill et al., 2018). A corollary to these conclusions is that 2-AG augmentation may represent a novel approach for the treatment of anxiety and stress-related neuropsychiatric disorders, however, there are some contradictory findings in this regard. Specifically, as noted above (**Figure 2**), 2-AG augmentation increased the acquisition of conditioned fear responses, increased the expression of conditioned fear (Llorente-Berzal et al., 2015), and impairs fear extinction (Hartley et al., 2016). Thus, there appears to be an emerging distinction between the effects of 2-AG augmentation on conditioned versus unconditioned fear behaviors, with only the latter being consistently reduced in response to pharmacological 2-AG augmentation.

A critical question that arises from these findings is how both augmenting and depletion of 2-AG results in similar fear extinction deficits. The fear promoting effects of 2-AG augmentation are absent in mice with GABA neuron-specific CB1 deletion, supporting the importance of 2-AG acting on GABAergic neurons to impair extinction and promote conditioned fear (Llorente-Berzal et al., 2015). The retrograde inhibition of GABA release may regulate important circuits within the basolateral amygdala thereby enhancing neuronal output to the central amygdala controlling freezing behavior. It is also important to point out that CB1 deletion from forebrain glutamatergic neurons itself impairs fear extinction (Llorente-Berzal et al., 2015). Based on these data, we propose the extinction-impairing and anxiogenic effects of 2-AG deficiency are due to reduced activity at CB1 on limbic glutamatergic terminals, which may be tonically suppressed by 2-AG signaling. In contrast, the fear promoting and extinction impairing effects of MAGL inhibition are mediated via 2-AG accumulation, synaptic spillover, and subsequent activation of CB1 on GABAergic neurons controlling freezing behavior. We hypothesize that these synapses may physiologically be under less tonic inhibition by 2-AG than glutamatergic CB1, thus 2-AG depletion does not significantly change CB1 activity on GABAergic cells and does not produce anxiolytic effects *per se*. A critical assumption in this model is the differential tonic inhibition of glutamate and GABA release mediated via 2-AG-CB1 signaling, an effect which remains to be tested experimentally. Additionally, that 2-AG augmentation reduces unconditioned anxiety suggests 2-AG may be acting on distinct neural circuits to affect excitation/inhibition balance to ultimately differentially affect conditioned versus unconditioned behaviors.

With regard to the therapeutic potential of eCB signaling, pharmacological AEA augmentation via inhibition of FAAH has also been demonstrated to have anxiolytic and anti-stress effects (Piomelli et al., 2006; Gunduz-Cinar et al., 2013a; Hill et al., 2018), and to facilitate extinction of conditioned fear in some models (Gunduz-Cinar et al., 2013b; Llorente-Berzal et al., 2015). Furthermore, we have recently demonstrated that FAAH inhibition can prevent the unconditioned anxiety associated with acute 2-AG depletion (Bedse et al., 2017). However, FAAH inhibition at the same dose used in Bedse et al. (2017) was unable to enhance fear extinction in mice treated with DO34, suggesting AEA cannot compensate for all aspects of DAGL inhibition. Furthermore, the addition of the CB1 partial agonist, THC was unable to overcome 2-AG-deficiency-induced impairments in fear extinction at dose that has mitigated anxiety like behaviors in non-fear conditioning paradigms (Bedse et al., 2017). In Bedse et al. (2017), when animals were treated with 0.3 mg/kg dose of THC + 50 mg/kg of DO34, they showed decreases in anxiety like behaviors versus animals treated with DO34 alone. In this study, we tested both 0.3 and 0.6 mg/kg doses and found no significance between doses in total freezing behavior or the ability to reverse fear extinction impairments caused by DAGL inhibition. Whether direct full CB1 agonists or CB1 positive allosteric modulators, or alternative doses of THC, could overcome DAGL inhibition-induced impairments in fear extinction is a critical open question in the field of cannabinoid therapeutics development. Lastly, our data might suggest the possibility that the impairment if fear extinction after DAGL inhibition is not mediated by 2-AG deficiency, but rather other mechanisms secondary to the widespread lipidomic changes induced by DAGL inhibition.

## Conclusion

By utilizing genetic and pharmacological approaches, we have demonstrated that DAGL activity plays an important role in fear extinction learning in both male and female mice. We have also shown that augmentation of AEA levels during fear extinction cannot reverse fear extinction deficits caused by disrupting the molecular mechanisms regulating 2-AG synthesis. Our data combined with data in previous studies highlights the intriguing paradoxical findings that both depletion and augmentation of 2-AG levels impairs fear extinction. There are some limitations to the present work that should be considered in the context of the above interpretations. For example, DAGL inhibition decreases levels of several monoacylglycerols in addition to 2-AG and reduces arachidonic acid levels (Shonesy et al., 2014; Ogasawara et al., 2016), both of which could affect physiology and behavior independent of 2-AG-mediated eCB signaling. In addition, both genetic and pharmacological approaches utilized here are systemic, therefore, the specific brain regions and circuits responsible for the behavioral effects of DAGL inhibition are not currently known. Despite these limitations, the present data provide a solid framework from which to test critical hypotheses regarding the potential therapeutic benefits of eCB modulating compounds on stress-related behavioral outcomes.

## Author Contributions

VC conducted the behavioral experiments with assistance from AG, DP, and PJ. VC and SP contributed to the research design, data interpretation, and analysis in the laboratory of SP. JU contributed to the generation of reagents in the laboratory of LM. VC and SP wrote the manuscript with input from all the authors.

## Conflict of Interest Statement

SP and LM receive research support from H. Lundbeck A/S. SP is a scientific consultant for Psy Therapeutics. The remaining authors declare that the research was conducted in the absence of any commercialor financial relationships that could be construed as a potential conflict of interest.

## Supplementary Material

FIGURE S1 DO34 treatment prior to extinction training mimics the effect of no fear extinction training. **(A)** Schematic diagram of the experimental paradigm. **(B)** (Far left panel) % freezing by C57BL/6J male mice during conditioning assay (Middle panels) % freezing during auditory cue during extinction training days 2 and 3. “No extinction” group remained in home cage during extinction trials day 2−3. (Far right panel) % freezing during extinction recall (*n* = 15 male mice per condition). *F*- and *P*-values and η^2^ for main effect of condition (extinction versus no extinction) shown in relevant panels. All values are presented as mean ± SEM.

FIGURE S2 THC does not reverse impaired extinction after DAGL inhibition. **(A)** Schematic diagram of the experimental paradigm. **(B)** (Far left panel) % freezing by C57BL/6J male mice during acquisition of cue-conditioned fear. (Middle panels) % freezing during auditory cue by mice during extinction training days 2 and 3 when DO34 (50 mg kg^−1^) was injected IP 2 h prior to trial, alone or with an additional injection of THC 30 min prior to extinction training (THC 0.6 mg kg^−1^) (Far right panel) % freezing during auditory cue during CS recall (*n* = 14 DO34 + THC-treated male mice, *n* = 13 DO34 + vehicle = treated male mice, *n* = 14 vehicle + vehicle-treated male mice). *F*- and *P*-values and η^2^ obtained by repeated measures two-way ANOVA representing effect of DAGL + THC treatment, DO34 + vehicle treatment, and vehicle only treated animals, prior to fear extinction training. A separate ANOVA was conducted to compare DO34 + THC and DO34 + vehicle-treated animals, and is shown. Colored dots corresponding to treatment legend delineates which treatments are represented in each analysis. All values are given as mean ± SEM. **(C)** In a separate experiment, there was no significant difference in total freezing behavior between two doses of THC (THC 0.3 and 0.6 mg kg^−1^) on extinction days 2 and 3. Significance values obtained by Student’s *t*-test of total freezing behavior during the entire trial.
