# Supplementary figures and images for: Inhibition of Diacylglycerol Lipase Impairs Fear Extinction in Mice
Source: Front Neurosci. 2018 Jul 31;12:479. doi: 10.3389/fnins.2018.00479 (PMC6080414; doi:10.3389/fnins.2018.00479)

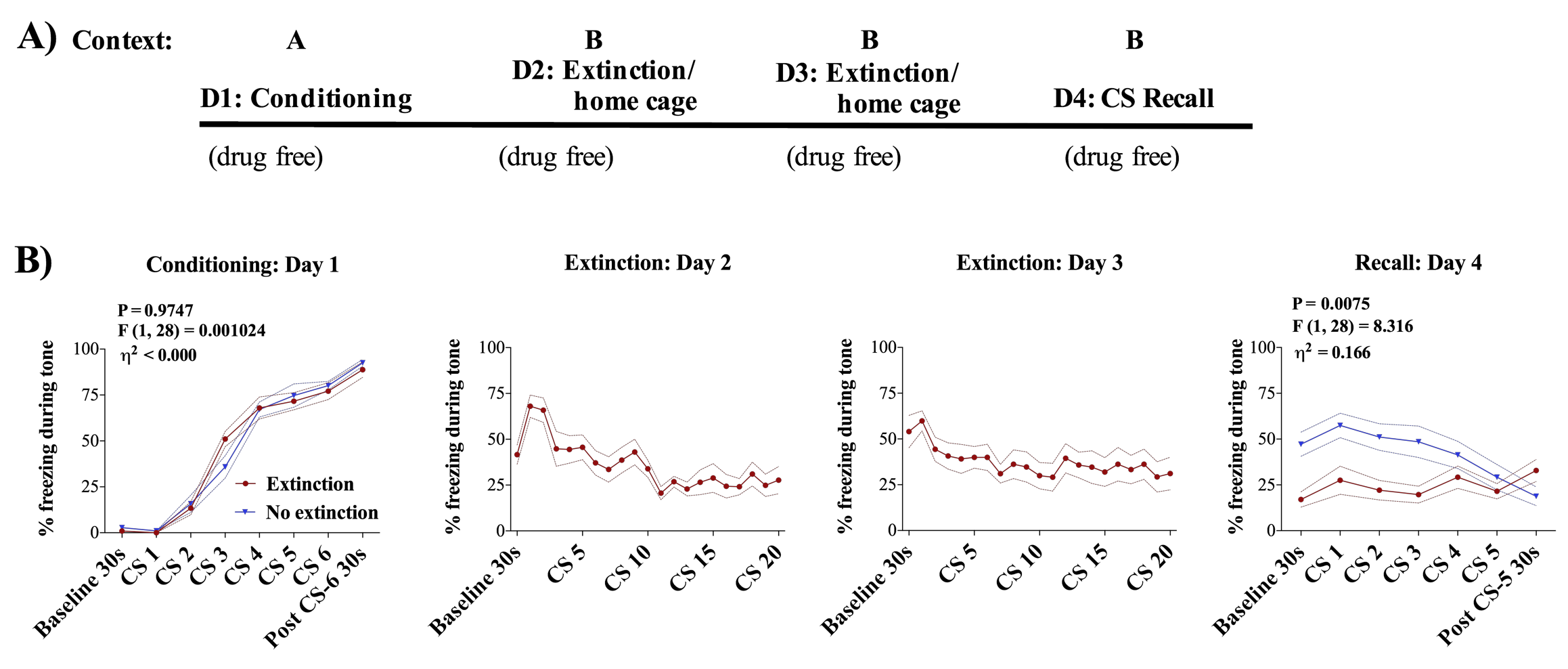

Supplement: FIGURE S1 — DO34 treatment prior to extinction training mimics the effect of no fear extinction training. (A) Schematic diagram of the experimental paradigm. (B) (Far left panel) % freezing by C57BL/6J male mice during conditioning assay (Middle panels) % freezing during auditory cue during extinction training days 2 and 3. “No extinction” group remained in home cage during extinction trials day 2−3. (Far right panel) % freezing during extinction recall (n = 15 male mice per condition). F- and P-values and η2 for main effect of condition (extinction versus no extinction) shown in relevant panels. All values are presented as mean ± SEM. [file Image_1.tiff]

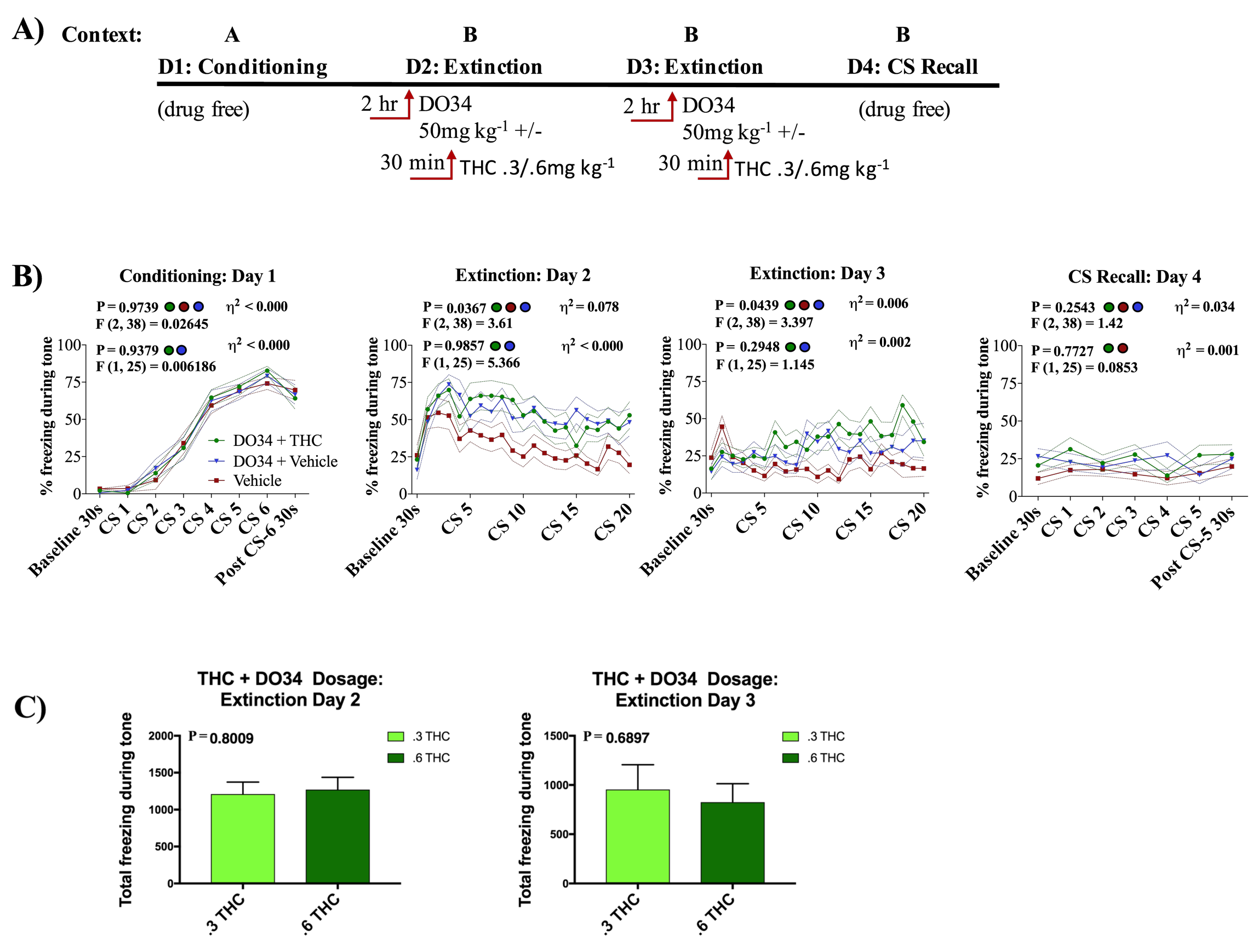

Supplement: FIGURE S2 — THC does not reverse impaired extinction after DAGL inhibition. (A) Schematic diagram of the experimental paradigm. (B) (Far left panel) % freezing by C57BL/6J male mice during acquisition of cue-conditioned fear. (Middle panels) % freezing during auditory cue by mice during extinction training days 2 and 3 when DO34 (50 mg kg−1) was injected IP 2 h prior to trial, alone or with an additional injection of THC 30 min prior to extinction training (THC 0.6 mg kg−1) (Far right panel) % freezing during auditory cue during CS recall (n = 14 DO34 + THC-treated male mice, n = 13 DO34 + vehicle = treated male mice, n = 14 vehicle + vehicle-treated male mice). F- and P-values and η2 obtained by repeated measures two-way ANOVA representing effect of DAGL + THC treatment, DO34 + vehicle treatment, and vehicle only treated animals, prior to fear extinction training. A separate ANOVA was conducted to compare DO34 + THC and DO34 + vehicle-treated animals, and is shown. Colored dots corresponding to treatment legend delineates which treatments are represented in each analysis. All values are given as mean ± SEM. (C) In a separate experiment, there was no significant difference in total freezing behavior between two doses of THC (THC 0.3 and 0.6 mg kg−1) on extinction days 2 and 3. Significance values obtained by Student’s t-test of total freezing behavior during the entire trial. [file Image_2.tiff]
